# Stress-induced dorsal striatal dopamine release is associated with changes in functional connectivity to the prefrontal cortex in nicotine-dependent individuals—a preliminary report

**DOI:** 10.3389/fnins.2026.1946892

**Published:** 2026-10-27

**Authors:** Siyan Fan, Su Jin Kim, Yasmin Zakiniaeiz, Jocelyn Hoye, Bart de Laat, Alaaddin Ibrahimy, Gelsina Stanley, Chiang-Shan R. Li, Kelly P. Cosgrove, Michelle Hampson, Evan D. Morris

**Affiliations:** 1 Department of Psychiatry, Yale School of Medicine, New Haven, CT, United States; 2 Radiology and Biomedical Imaging, Yale School of Medicine, New Haven, CT, United States; 3 Department of Biomedical Engineering, Yale School of Engineering and Applied Science, New Haven, CT, United States

**Keywords:** functional connectivity (FC), multimodal neuroimaging, nicotine dependence (ND), PET-MRI imaging, striatal dopamine release

## Abstract

**Introduction:**

The neural mechanism linking stress to addiction pathophysiology remains unclear. A proposed pathway involves stress-induced dopamine (DA) transmission impairing inhibitory control, leading to relapse. Lipopolysaccharide (LPS), a biological stressor inducing systemic inflammation, amplifies DA release. Our prior study demonstrated this effect in smokers. Using the same cohort, we investigated whether DA transmission mediates acute stress effects and impacts striatal-prefrontal functional connectivity (FC) in brain regions governing reward, inhibition, and decision-making.

**Methods:**

Using a within-subject, multimodal design, we collected PET and resting-state functional MRI (rs-fMRI) scans in 8 smokers (6 females; mean age: 32 ± 3.6 years). Stress was induced using LPS; plasma cortisol indexed level of stress. DA release was measured in striatal subregions using [^11^C]raclopride PET before and after methylphenidate administration, while striatal–prefrontal functional connectivity was assessed using seed-based rs-fMRI. Each participant completed three PET scans (one baseline and two post-methylphenidate scans following LPS or placebo [PCBO] pretreatment) and two rs-fMRI scans (LPS and PCBO), resulting in a total of 24 PET scans and 16 rs-fMRI scans.

**Results:**

LPS elevated cortisol and DA release in striatum compared to Placebo (*p* < 0.05). Cortisol increases correlated with dorsal striatal DA release (*r* = 0.708, *p* = 0.04). FC analyses revealed enhanced FC between dorsal striatum and ventral medial prefrontal cortex (vmPFC) (*p* < 0.001, FDR-corrected), and between whole striatum and dorsal lateral PFC (dlPFC) (*p* < 0.001, FDR-corrected). Nonlinear associations showed FC initially increased with stress but plateaued at higher stress levels.

**Conclusion:**

Stress-induced changes in DA release within dorsal striatum influence stress-related alterations in frontal-striatal FC. Our present findings in a limited cohort lead us to hypothesize more broadly that stress alterations in frontal-striatal FC reflect adaptation, whereas excessive stress may disrupt circuits, potentially contributing to addictive behaviors such as smoking. While the present pilot study demonstrates the potential value of PET and rs-fMRI to connect neurotransmitter action to downstream cortical activity future work will be essential to confirm the reproducibility and relevance of the observed associations.

## 1 Introduction

According to the Centers for Disease Control and Prevention, cigarette smoking is responsible for more than 480,000 deaths annually and costs the United States over $600 billion in a single year (Cornelius et al., 2023). Research indicates that stress is a significant factor in addictive behaviors (Al'Absi, 2011). Further, recent global reports have emphasized increasing exposure to chronic societal stressors and instability, which have been associated with worsening mental health outcomes and rising rates of substance use and behavioral addictions worldwide (Dresp-Langley, 2023; Wyatt, 2025). Nonetheless, the exact neural mechanism(s) linking stress, reward-related processes and nicotine addictive behavior remain unclear.

Dopaminergic signaling plays a central role in integrating stress responses with motivational and reward-related processes, and dysregulation of this system has been implicated in vulnerability to addiction (Dresp-Langley, 2023; Wyatt, 2025; Volkow et al., 2004). This is especially evident in the striatum including both ventral and dorsal striatal subdivisions, a subcortical brain region integral to reward and motivation (Volkow et al., 2011). Further, stress-related striatal dopamine (DA) alterations have often been linked to deficits in reward-circuit functioning in addiction and stress response (Baik, 2020), and we have previously shown that stress can amplify striatal DA release, potentially supporting this mechanistic link (Zakiniaeiz et al., 2022; Petrulli et al., 2017).

Prefrontal brain regions, such as the ventromedial prefrontal cortex (vmPFC) and dorsolateral prefrontal cortex (dlPFC), play critical roles in decision-making, reward processing, and impulse control (Miller and Cohen, 2001). Dysfunction in these areas has also been strongly linked to the pathology of addiction (Goldstein and Volkow, 2011). Functional magnetic resonance imaging (fMRI) studies consistently show that smokers exhibit reduced activation in the prefrontal cortex when exposed to smoking-related cues, compared to neutral cues, suggesting a diminished ability to regulate cravings and control the urge to smoke (Wilson et al., 2004).

Impaired connectivity between the striatum and vmPFC contributes to poor decision-making and dysregulation of reward circuitry, limiting the ability to balance long-term consequences against immediate rewards—a hallmark of addiction (Baik, 2020; Blum et al., 2022; Koob and Volkow, 2010; Volkow et al., 2011). The brain prioritizes drug-related rewards over health outcomes (Volkow and Baler, 2015). Similarly, dysfunction in the striatum-dlPFC pathway disrupts self-control and impulse regulation, leading to weakened inhibitory control and greater difficulty in resisting cravings and breaking habitual drug use (Koob and Volkow, 2010). Striatal functional connectivity to these PFC regions has been found to be associated with stress-related psychopathology (Hanson et al., 2018; Seo et al., 2024; Zhukovsky et al., 2024).

While extensive evidence supports stress-related DA effects on corticostriatal circuits (Baik, 2020; Koob and Volkow, 2010), how acute stress–induced striatal DA release influences frontostriatal functional connectivity in nicotine addiction remains incompletely characterized. To address these gaps in the current understanding, we conducted a multimodal investigation in smokers using both positron emission tomography (PET) imaging and resting-state functional magnetic resonance imaging (rs-fMRI) in a within-subject design.

Previously, we administered an acute intravenous dose of lipopolysaccharide (LPS) as a laboratory-based model of stress. LPS activates the innate immune system and induces physiological and neuroendocrine responses resembling those elicited by psychosocial stressors, including elevations in pro-inflammatory cytokines, activation of the hypothalamic–pituitary–adrenal axis, increased cortisol, elevated heart rate and blood pressure, and mood and affect changes (see Supplementary Table S1). Importantly, we have demonstrated that LPS modulates striatal DA signaling in humans (Zakiniaeiz et al., 2022; Petrulli et al., 2017). Using [^11^C]raclopride PET, we showed that LPS amplifies methylphenidate (MP)-induced DA release in the striatum of healthy adults, significantly increasing MP-induced DA release in the caudate and putamen—paralleling DA reactivity observed under psychosocial stress (Petrulli et al., 2017). A subsequent study was conducted in tobacco smokers in comparison to the same cohort of healthy individuals and found LPS-induced inflammation significantly amplifies MP-induced striatal DA elevation in an addicted population as well. These findings suggest that LPS not only induces a physiological stress profile, but also engages central DA systems involved in reward processing and salience — systems highly relevant to stress-related psychopathology. Together, this work provides strong justification for using LPS as a controlled, reproducible, and mechanistically informative model of stress-related immune-brain interactions (Zakiniaeiz et al., 2022; Petrulli et al., 2017).

In the current study, cortisol levels measured during MR imaging were used as a biological marker of stress induced by LPS administration. We investigated the effects of stress on striatal dopamine release, focusing on pre-defined dorsal, ventral, and whole striatum regions, as assessed by PET imaging, and explored how changes in DA release influenced striatal functional connectivity to the vmPFC and dlPFC. We hypothesized that, relative to placebo (PCBO): (1) Stress would induce an increase in cortisol levels in smokers; (2) Stress-induced DA release would be elevated in all striatal regions; (3) Increases in cortisol levels would correlate with elevations in striatal DA release; (4) Stress-induced changes in functional connectivity from the striatal regions to the vmPFC and dlPFC would be detectable; (5) Elevations in striatal DA release would be associated with changes in striatal connectivity to the vmPFC and dlPFC.

## 2 Materials and methods

The study received approval from Yale University’s Human Investigation Committee, Radioactive Drug Research Committee, and Radiation Safety Committee. Informed consent was obtained from all participants before any study procedures were performed.

### 2.1 Subjects

Both male and female participants were eligible for inclusion if they were aged 18–40 years; able to read and write English; able to provide voluntary informed consent; and medically and psychiatrically healthy, with no current symptoms of medical or psychiatric illness. Participants met DSM-IV criteria for nicotine dependence as assessed by the Structured Clinical Interview for DSM Disorders (SCID) (First et al., 2015) and had a Fagerström Test for Nicotine Dependence (FTND) score greater than 3. All participants were daily smokers who reported smoking more than 7 cigarettes per day for at least 1 year and were not actively seeking smoking cessation. Smoking status was biochemically verified at intake by carbon monoxide (CO) levels greater than 10 parts per million (ppm) and urine cotinine concentrations exceeding 100 ng/mL. For exclusion criteria, all participants were free from significant medical conditions based on physical examination and did not meet DSM-5 (First et al., 2015) criteria for any current or past psychiatric or substance use disorder, aside from nicotine dependence. Pregnancy and lactation were exclusionary. All participants met these inclusion and exclusion criteria at the time of enrollment.

Eight tobacco smokers (6 females), with an average age of 32 ± 4.0 years (mean ± SE), participated in this study. On average, they smoked 15 ± 2.9 cigarettes daily over a span of 16 ± 4.2 years, with Fagerström Test for Nicotine Dependence (FTND) (Etter et al., 1999) scores averaging 5.8 ± 0.8, indicating moderate dependence (see Table 1). Smoking status was verified on the intake day by measuring carbon monoxide (CO) levels, which exceeded 11 parts per million (ppm), and by checking urine cotinine levels—the main nicotine metabolite—exceeding 150 ng/mL using NicAlert cotinine test strips (Nymox Pharmaceutical). On the day of the scan, each participant’s overnight abstinence from smoking was confirmed with CO levels below 10 ppm or at 50% or less of their intake day level (see Table 2).

**Table 1:** Subject demographics.

Characteristics	Smoker
Sex (N female)	6
Age	33 ± 4.0
Cigarettes/day	15 ± 2.9
Years smoked	16 ± 4.2
FTND	5.8 ± 0.8
Scan Order (N PCBO first)	6
Bodyweight (kg)	75 ± 6.0
LPS dose (ng)	60 ± 4.7
LPS dose/bodyweight (ng/kg)	0.80 ± 0.0006
MP/bodyweight (mg/kg)	0.56 ± 0.04
Injected Activity (mCi)	19 ± 0.61
Δ Injected Mass (PCBO‚ Baseline; μg)	0.34 ± 0.88
Δ Injected Mass (LPS‚ Baseline; μg)	0.05 ± 0.57

Δ Injected Mass refers to the difference in the mass of the radiotracer injected between the conditions described. FTND, Fagerström Test for Nicotine Dependence. Mean ± SE shown.

**Table 2:** Changes in striatal functional connectivity to PFC and associations between changes in DA release and striatal functional connectivity to PFC (all clusters reported in the table corrected for false discovery rate cluster *p*-value of 0.05).

Cluster	Lateralization	Cluster size	MNI coordinates		
Changes in striatal functional connectivity to prefrontal cortex					
			*x*	*y*	*z*
To dorsal lateral prefrontal cortex	Left	575	−12	54	38
To ventral medial prefrontal cortex	Left	383	−8	48	−10
Associations between changes in dopamine (DA) release and changes in changes in striatal functional connectivity to prefrontal cortex					
Between striatal DA release and striatal connectivity to dorsal lateral prefrontal cortex	Right	263	4	44	28
Between dorsal Striatal DA release and dorsal striatal connectivity to ventral medial prefrontal cortex	Left	42	2	32	−20
Between ventral Striatal DA release and ventral striatal connectivity to ventral medial prefrontal cortex	Right	80	−8	60	12

### 2.2 Study design and experimental protocol

#### 2.2.1 Overview

This study employed a within-subject, double-blind, randomized, placebo-controlled design combining PET and resting-state functional magnetic resonance imaging (rs-fMRI). Each participant completed PET and MRI sessions under both LPS and placebo (PCBO) conditions (Figure 1).

**Figure 1:**
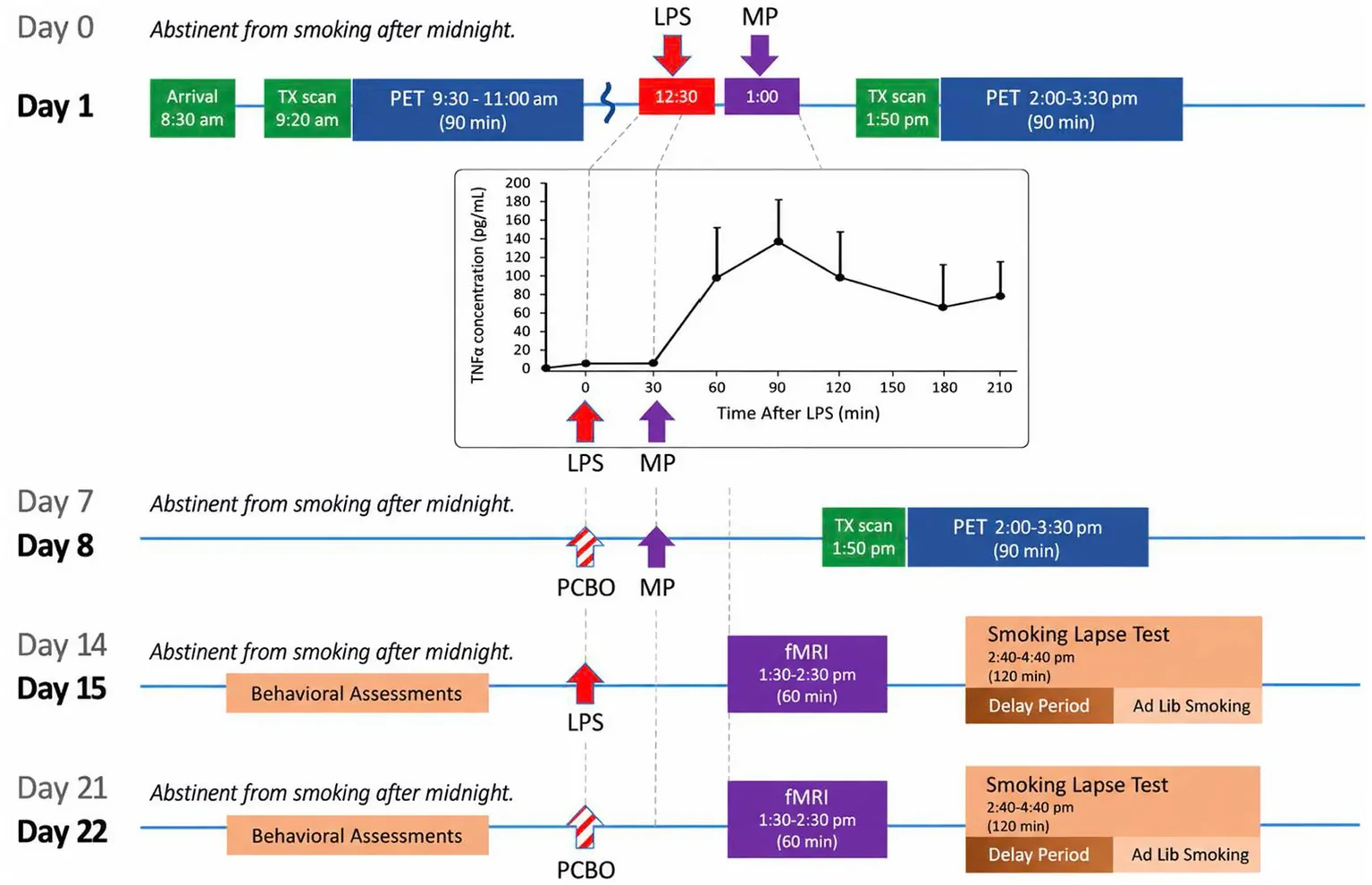
Study design: subjects participated in scans and/or lab sessions on 4 separate days. Two days involve LPS but one of them includes MP as well (which lessens sickness symptoms). Study design and experimental timeline. Participants completed four experimental study days involving positron emission tomography (PET), resting-state functional magnetic resonance imaging (rs-fMRI), and/or behavioral assessments. PET and rs-fMRI procedures were conducted under lipopolysaccharide (LPS) or placebo (PCBO) conditions, with methylphenidate (MP) administered during PET sessions as indicated. Participants abstained from smoking after midnight before each imaging or behavioral study day. On PET study days (Days 1 and 8), participants underwent [^11^C]raclopride PET imaging. On Day 1, a baseline PET scan was acquired before LPS administration, followed by MP administration and a post-LPS/MP PET scan. On the alternate PET study day, participants received PCBO followed by MP and underwent an MP challenge PET scan. LPS or PCBO was administered at *t* = 0, and MP was administered 30 min later. Post-treatment PET scanning commenced 60 min after MP administration (i.e., 90 min after LPS/PCBO), corresponding to the period of peak peripheral inflammatory response. LPS and PCBO conditions were administered using a randomized, double-blind, within-subject design. Resting-state fMRI scans were conducted on separate study days (Days 15 and 22), approximately 1 week apart, under LPS and PCBO conditions. The LPS/PCBO dose and administration procedures matched those used on the PET study days; however, MP was not administered during the fMRI sessions. Behavioral assessments and smoking lapse tests were conducted in conjunction with the fMRI study days to assess the effects of inflammatory stress on frontostriatal functional connectivity and smoking behavior independent of the MP challenge. PET, positron emission tomography; rs-fMRI, resting-state functional magnetic resonance imaging; LPS, lipopolysaccharide; PCBO, placebo; MP, methylphenidate.

#### 2.2.2 PET scan days

Each participant underwent a structural MRI scan on a Prisma 3 T MR scanner (Siemens/CTI, Knoxville, TN, USA) to support anatomical localization. Participants also completed PET imaging across two separate scan days (Figure 1).

On each PET scan day, participants completed either (i) two PET scans—a baseline scan followed by a methylphenidate (MP) + LPS/placebo (PCBO) scan—or (ii) a single MP + LPS/PCBO scan. The baseline scan consisted of a 90-min [^11^C]raclopride PET acquisition.

For the MP + LPS/PCBO scans, participants received an intravenous pre-treatment of either lipopolysaccharide (LPS; 0.8 ng/kg) or saline placebo (PCBO) at time t = 0 min. Thirty minutes later, participants received an oral dose of MP (40 mg). PET scanning commenced 60 min after MP administration, corresponding to 90 min after the LPS/PCBO pre-treatment. The timing of LPS administration was selected to coincide with peak plasma levels during PET acquisition, consistent with prior work (Petrulli et al., 2017; Sandiego et al., 2015).

Participants returned for a second PET session at least 1 week later and underwent the same procedure (LPS/PCBO pre-treatment, MP administration, and MP + LPS/PCBO scan) under the alternate pre-treatment condition. Pre-treatment order was randomized and double-blinded. Six of the eight participants completed their two PET sessions 8 days apart, with a mean inter-session interval of 22 days (SD = 28 days). For most participants (7 of 8), the baseline PET scan occurred during the first PET session. In addition, six of the eight participants received the MP + PCBO condition during their first MP challenge PET scan. Minor deviations from the original randomization occurred due to occasional scan cancellations; however, no significant order effects were observed (Zakiniaeiz et al., 2022).

The selected MP dose was based on prior studies demonstrating robust reductions in [^11^C]raclopride binding (approximately 10–30%), reflecting dopamine release (Zakiniaeiz et al., 2022; Volkow et al., 2001; Volkow et al., 2002). The LPS dose was informed by previous fMRI and PET studies reporting effects on brain activity (Eisenberger et al., 2010; Hannestad et al., 2012)and altered dopamine responses to MP (Petrulli et al., 2017). Additional details of the PET study design have been described previously (Zakiniaeiz et al., 2022).

#### 2.2.3 MR scan days

Each participants’ resting-state functional MRI scans were conducted on two separate occasions, distinct from PET scan days, 1 week apart, under either the LPS or PCBO condition, using a Prisma 3 T MR scanner (Siemens/CTI, Knoxville, TN, USA) (Figure 1). The timing and dosage for LPS and PCBO administration were kept consistent with those used on the PET scan days. No oral MP was administered to participants on the MRI scan days.

### 2.3 Blood sampling

Each subject’s venous blood samples (10 mL each) were collected at 30, 90, 120, 150, 180 and 210 min after LPS/PCBO administration to assess cortisol levels. Blood samples were centrifuged for 10 min, and the plasma was stored at −80 °C. Plasma samples were analyzed for cortisol levels at the Yale Center for Clinical Investigations (YCCI) Lab in New Haven, CT, USA.

### 2.4 Image analysis–data acquisition

#### 2.4.1 PET imaging

Participants were allowed to have a light, caffeine-free breakfast before the scan. They were instructed to avoid anti-inflammatory medications for at least 3 days prior to each scan session. An intravenous catheter was inserted to enable ^11^C-raclopride injection and periodic blood sampling. Medical staff monitored blood pressure, heart rate, and temperature before, during, and after the scan to ensure participants’ safety. Details on PET imaging parameters, procedures, methods, and analysis have been described previously (Zakiniaeiz et al., 2022).

#### 2.4.2 MRI imaging

Similar to PET scan days, participants were allowed to have a light, caffeine-free breakfast before the scan. Medical staff monitored participants’ blood pressure, heart rate, and temperature before, during, and after the scan to ensure their well-being.

All participants were scanned using a 64-channel head coil. Each session commenced with a localizing scan, followed by a high-resolution anatomical scan using an MPRAGE sequence with the following parameters: repetition time (TR) of 2,530 ms, echo time (TE) of 2.77 ms, inversion time (TI) of 1,100 ms, a flip angle of 7°, and a resolution of 1 × 1 × 1 mm. A set of 34 axial-oblique T1-weighted slices, each 4 mm thick, was aligned with the 13th slice positioned at the anterior commissure–posterior commissure line. Functional images were acquired from the same slice locations as the T1-weighted anatomical scans, using a T2*-sensitive gradient-recalled, single-shot echo-planar imaging sequence with TR = 1,000 ms, TE = 30 ms, flip angle = 60°, field of view (FOV) = 220 mm, and a matrix size of 64^2^. Participants completed two resting-state scan (165 volumes, lasting 5 min and 30 s). During this scan, they were instructed to keep their eyes open while focusing on a crosshair.

### 2.5 Subject level image processing

#### 2.5.1 MR imaging

##### 2.5.1.1 Preprocessing

Preprocessing was performed using the BioImage Suite software package.^1^ The functional imaging data underwent motion correction using SPM12’s algorithm.^2^ Participants with an average framewise displacement exceeding 0.3 mm were excluded (Greene et al., 2018; Vanderwal et al., 2021); no participants exceeded this threshold, and therefore no participants were excluded based on head motion. For each participant, the high-resolution T1-weighted structural image was segmented into gray matter, white matter, and cerebrospinal fluid (CSF) tissue compartments using SPM12. The structural image was co-registered to the functional images, and the resulting transformation was applied to the tissue masks to align them with the resting-state fMRI data. Several nuisance variables were regressed out from the time series, including the six motion parameter time courses, mean white matter signal, mean cerebrospinal fluid signal, and up to a third-order polynomial drift (polynomial adjustments were specific to each voxel, removing low-frequency fluctuations outside the range of interest). Temporal smoothing was applied to the data using a Gaussian filter with a cutoff frequency of 0.104 Hz, and the analysis was restricted to gray matter voxels.

##### 2.5.1.2 Defining striatal seed regions and cortical regions of interest

The Oxford-GSK-Imanova Striatal Connectivity Atlas was used as the whole striatum mask. The ventral striatum mask was created by using automated anatomical labeling (AAL) (Tzourio-Mazoyer et al., 2002). Because it is not included in AAL, the dorsal striatum mask was obtained by subtracting the ventral striatum mask from the whole striatum mask. The bilateral dlPFC and vmPFC regions of interest were defined using Brodmann areas (9 and 46 for dlPFC; 10 and 11 for vmPFC) from the Talairach atlas in MNI space, as provided by the BioImage Suite software (Lacadie et al., 2008).

##### 2.5.1.3 Connectivity analysis

Resting-state functional images from the LPS and PCBO conditions were coregistered to each participant’s high-resolution T1-weighted structural image, providing a common subject-specific anatomical space and accounting for differences in image origin and orientation across imaging sessions. The three striatal regions (dorsal, ventral and whole striatum) were mapped to each participant’s individual space, and seed connectivity maps were generated in BioImage Suite for each participant. A seed map was created by averaging time courses across voxels in each seed region to create a reference time-course. This reference time-course was then correlated with the time-course of each voxel in the brain to yield a map of r values which was then transformed to z values using Fisher’s *r*-to-*z* transformation. Each individual’s striatal seed-maps in the PCBO condition were subtracted from his/her striatal seed-maps (for the same striatal seed) in the LPS condition.

##### 2.5.1.4 Transform to common space

Individual subject seed maps were aligned to the coordinate space of the Colin brain (Holmes et al., 1998) through a series of three registration steps: (i) a linear rigid transformation aligning the functional data with the axial-oblique anatomical data obtained during the same scanning session, (ii) a linear rigid transformation of the axial-oblique anatomical data to the subject’s MPRAGE image, and (iii) a nonlinear registration of the MPRAGE image to the Colin brain (Papademetris et al., 2001). All registrations were visually inspected to confirm accuracy.

#### 2.5.2 PET imaging

##### 2.5.2.1 Preprocessing

The preprocessing pipeline has been described previously in details (Zakiniaeiz et al., 2022).

Briefly, PET emission data were corrected for attenuation, scatter, dead time, detector sensitivity, and random events and reconstructed using ordered-subset expectation maximization (OSEM; 2 iterations, 30 subsets). HRRT images were harmonized by down-sampling the voxel dimensions and applying an isotropic Gaussian filter (4.5-mm full width at half maximum [FWHM]). The resulting PET images were coregistered to each participant’s structural MRI and subsequently nonlinearly registered to a Montreal Neurological Institute (MNI) template in common space using BioImage Suite.

##### 2.5.2.2 Change in dopamine release

Regional time-activity curves for the cerebellum, whole striatum, dorsal striatum, and ventral striatum were generated by using the 3 striatal masks (see above sub-section of 2.5.1.2). These curves were fitted using the simplified reference tissue model (Lammertsma and Hume, 1996), with the cerebellum as the reference region, to calculate non-displaceable binding potential (BPND). Change in BPND (ΔBPND) was calculated between baseline and post-MP for both LPS and PCBO pre-treatment conditions. Percent change in BPND is a widely used approach with several dopamine radiotracers, including [^11^C]raclopride, [^18^F]fallypride, [^11^C]PHNO, and [^11^C]FLB457 PET, to quantify the effect of alteration of endogenous dopamine (Martinez et al., 2003; Okita et al., 2016; Whitton et al., 2020; Zakiniaeiz et al., 2019).

### 2.6 Group level analysis

#### 2.6.1 Dopamine elevation (PET imaging data)

To examine differences in MP-induced dopamine increase, a repeated-measures ANOVA (rmANOVA) was conducted with condition (MP + LPS vs. MP + PCBO) as a within-subjects factor on BPND values from predefined regions of interest (ROIs), including the whole striatum, ventral striatum, and dorsal striatum. Normality of ΔBPND values was confirmed using Kolmogorov–Smirnov and Shapiro–Wilk tests, as normality is a key assumption for the rmANOVA model (see additional details in our previous publication) (Zakiniaeiz et al., 2022).

#### 2.6.2 Connectivity (MR imaging data)

We applied an ROI-based approach using the MarsBaR toolbox in SPM^3^ for the below group analyses.

##### 2.6.2.1 Changes across conditions (LPS-PCBO)

Each individual’s striatal seed-maps in the PCBO condition were subtracted from his/her striatal seed-maps (for the same striatal seed) in the LPS condition. Voxel-wise one-sample t-tests were performed on subjects’ subtracted striatal seed-maps (LPS – PCBO) to examine changes in functional connectivity across conditions to the seed. Significance was assessed within the vmPFC and dlPFC using false discovery rate (FDR) with *p* < 0.05 small volume corrected threshold. The average change within significant clusters in these regions was extracted using the MarsBaR toolbox in SPM.

##### 2.6.2.2 Relationship between seed-connectivity and dopamine elevation

Voxel-wise regression analyses were conducted in SPM to examine the relationship between an individual’s subtracted striatal seed-maps (LPS – PCBO) to the vmPFC and dlPFC, and changes in ΔBPND (LPS – PCBO). Significance was assessed within the vmPFC and dlPFC using FDR with a *p* < 0.05 small volume corrected threshold.

### 2.7 Statistical analyses

All statistical analyses were performed using SPSS Statistics v22 (IBM). A significance threshold of *p* < 0.05 was used, with *p* < 0.1 considered a trend toward significance.

#### 2.7.1 Changes in cortisol levels across conditions

Paired t-tests were conducted to obtain changes in peak cortisol levels (LPS – PCBO), using averaged cortisol levels across the 120- and 150-min timepoints (per individual), as this window captures the peak LPS-induced effect.

#### 2.7.2 Correlation analyses between cortisol levels, dopamine elevation and connectivity

Pearson correlation analyses were first carried out to examine the relationship between changes (LPS – PCBO) in mean cortisol levels (averaged across the 120- and 150-min timepoints) and changes in striatal DA release (dorsal, ventral and full striatum).

Pearson correlation analyses were subsequently conducted to examine the relationships between changes in connectivity in the ROIs (where the LPS-PCBO change in striatal connectivity was significant, see 2.6.2.1) and changes in cortisol levels, as well as between ROI-striatal connectivity changes and striatal DA release.

## 3 Results

### 3.1 Blood analysis

Mean plasma MP levels were not different at any timepoints between MP + LPS and MP + PCBO conditions. Mean plasma cortisol levels were not different at 0, 30, 60, 90, 180, 240 min post-LPS injection. Mean cortisol levels were significantly higher at 150 min and trending higher at 120 min post-LPS injection for the MP + LPS compared to MP + PCBO condition (*p <* 0.05 or *p <* 0.1); (Figure 2).

**Figure 2:**
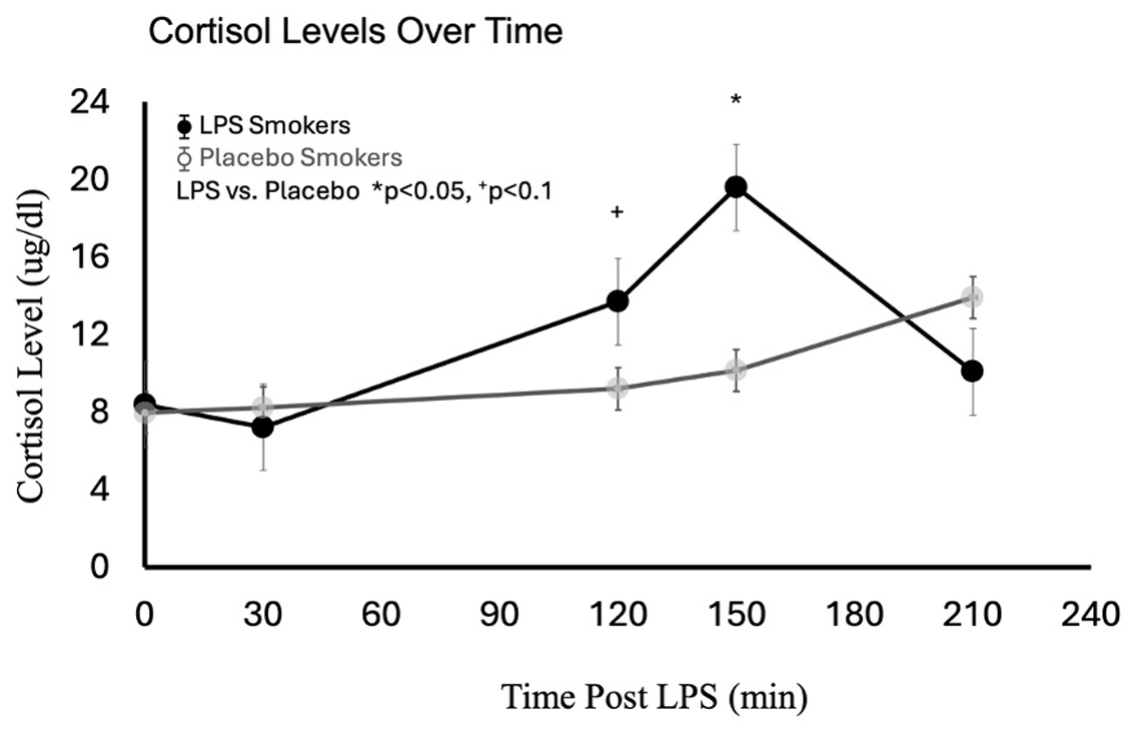
Cortisol levels over time. Plasma cortisol levels (ug/dl) at 0, 30, 60, 90, 120, 180 and 210 min post LPS injection. Mean an SE error bars shown. Plasma cortisol levels following LPS and placebo administration. Plasma cortisol levels (μg/dL) were measured at 0, 30, 120, 150, and 210 min following lipopolysaccharide (LPS) or placebo administration. Data are presented as mean ± standard error (SE). *p* < 0.05 and ^+*p* < 0.10 for LPS versus PCBO. LPS, lipopolysaccharide; SE, standard error.

#### 3.1.1 Dopamine elevation

rmANOVA showed an effect of condition (MP + LPS vs. MP + PCBO; *F*(1, 7) = 8.10, *p* = 0.0025, ηp^2^ = 0.54) in the whole striatum whereby dopamine elevation was significantly higher under the MP + LPS (ΔBPND = 18 ± 2.9% (Mean ± SE)) condition compared to the MP + PCBO (ΔBPND = 13 ± 2.7% (Mean ± SE)) condition. In ventral striatum, an effect of condition was observed, *F*(1, 7) = 11.34, *p* = 0.0035, ηp2 = 0.62; a similar effect was observed in the dorsal striatum, *F*(1, 7) = 8.02, *p* = 0.0005, ηp2 = 0.53 (see more details in our previous publication Zakiniaeiz et al., 2022). Because the striatal regions of interest were defined *a priori*, no correction for multiple comparisons was applied. Nevertheless, the dorsal striatum finding was significant after Bonferroni correction for three striatal sub-regions (*α* = 0.017).

#### 3.1.2 Correlation between changes in plasma cortisol levels and dorsal striatal dopamine elevation

We observed a positive correlation between changes in mean plasma cortisol levels and changes in dorsal striatal dopamine releases (*r* = 0.708, *p* = 0.04; Figure 3).

**Figure 3:**
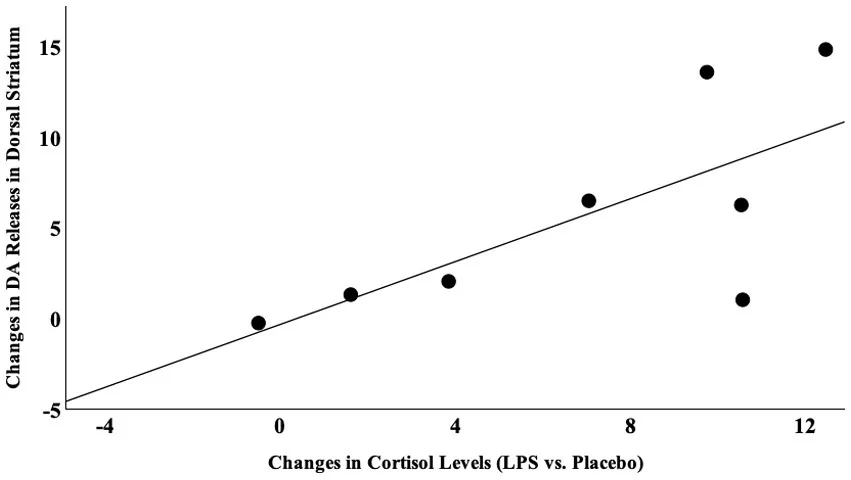
A positive correlation between changes in mean plasma cortisol levels and changes in dorsal striatal dopamine releases (*r* = 0.708, *p* = 0.04). Association between changes in plasma cortisol levels and dorsal striatal dopamine release. Changes in mean plasma cortisol levels following lipopolysaccharide (LPS) relative to placebo were positively correlated with changes in dorsal striatal dopamine (DA) release (*r* = 0.708, *p* = 0.04). The solid line represents the fitted linear regression line. LPS, lipopolysaccharide; DA, dopamine.

#### 3.1.3 Enhanced striatal functional connectivity to PFC

A within-subject comparison between LPS and PCBO conditions revealed significantly enhanced functional connectivity from the striatum and dorsal striatum to the dlPFC and vmPFC, respectively under LPS (FDR corrected, both at *p* < 0.001) (Figures 4A,B). There was a trend toward negative correlation between changes in mean plasma cortisol levels and changes in dorsal striatal functional connectivity to the vmPFC (*r* = −0.638, *p* = 0.089), as shown in Figure 4C for illustrative purposes.

**Figure 4:**
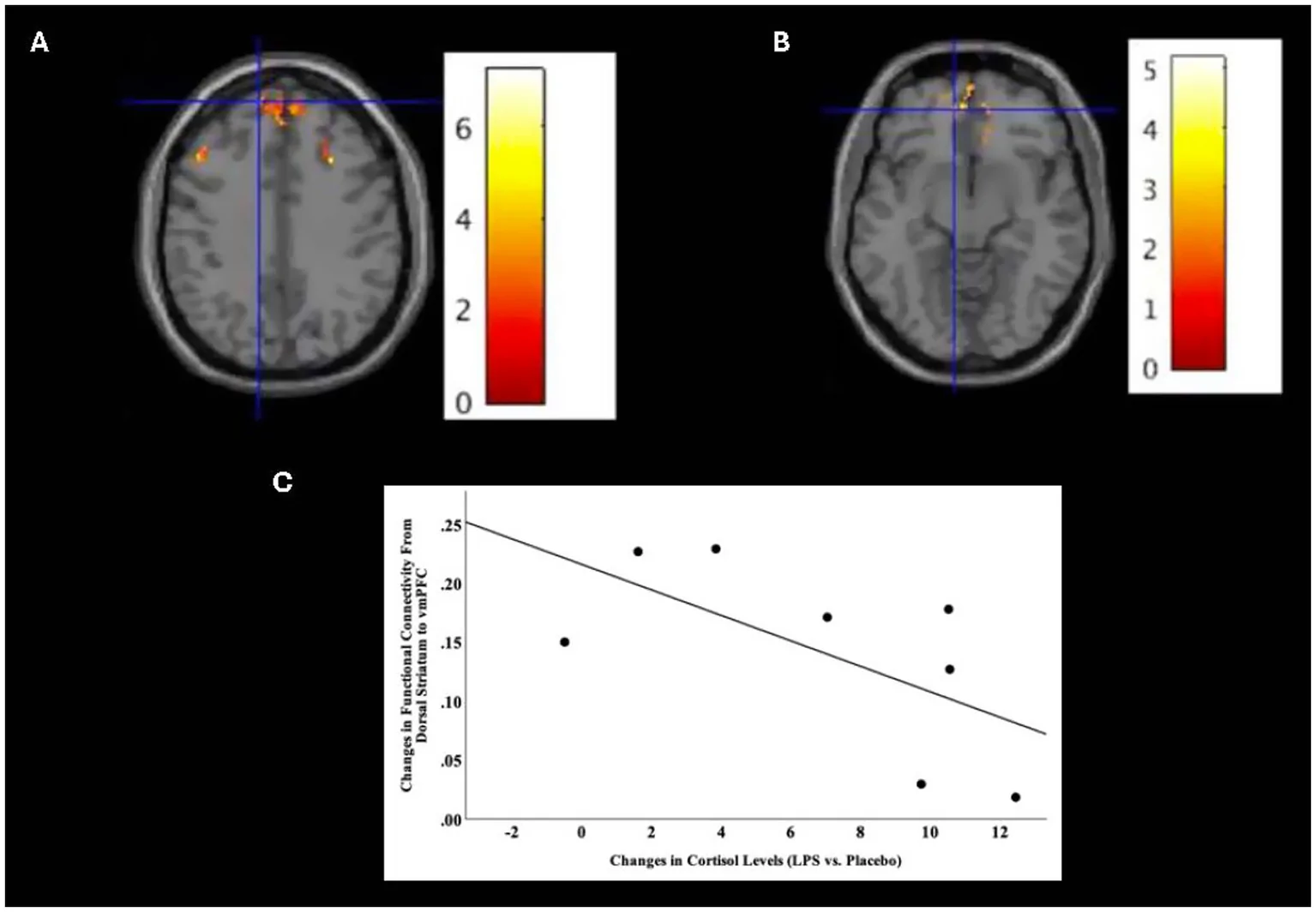
Enhanced striatal–prefrontal functional connectivity and its association with cortisol **(A)** Increased striatal functional connectivity with the dorsolateral prefrontal cortex. Region-of-interest analysis showing significantly increased striatal functional connectivity with the dorsolateral prefrontal cortex (dlPFC; false discovery rate [FDR]-corrected *p* < 0.005). Crosshairs indicate the peak Montreal Neurological Institute (MNI) coordinates (*x* = −12, *y* = 54, *z* = 38). **(B)** Increased dorsal striatal functional connectivity with the ventromedial prefrontal cortex. Region-of-interest analysis showing significantly increased dorsal striatal functional connectivity with the ventromedial prefrontal cortex (vmPFC; false discovery rate [FDR]-corrected *p* < 0.05). Crosshairs indicate the peak Montreal Neurological Institute (MNI) coordinates (*x* = −8, *y* = 48, *z* = −10). **(C)** Association between changes in plasma cortisol and dorsal striatum–ventromedial prefrontal cortex functional connectivity. Changes in mean plasma cortisol levels following lipopolysaccharide (LPS) relative to placebo showed a trend-level negative association with changes in functional connectivity between the dorsal striatum and ventromedial prefrontal cortex (vmPFC; *r* = −0.638, *p* = 0.089). The solid line represents the fitted linear regression line.

#### 3.1.4 Associations between changes in striatal dopamine releases and striatal connectivity

Significant correlations were observed between changes in striatal dopamine releases and striatal connectivity (FDR corrected, *p* < 0.05). We found that elevation of dopamine in the whole striatum was associated with less functional connectivity from the striatum to the dlPFC (FDR corrected, *p* = 0.001) (Figure 5A). In addition, elevations in dopamine in the ventral and dorsal striatum were associated with reduced functional connectivity between the respective striatal regions and the vmPFC (FDR-corrected *p* = 0.001 for the dorsal striatum and *p* = 0.003 for the ventral striatum) (Figures 5B–D).

**Figure 5:**
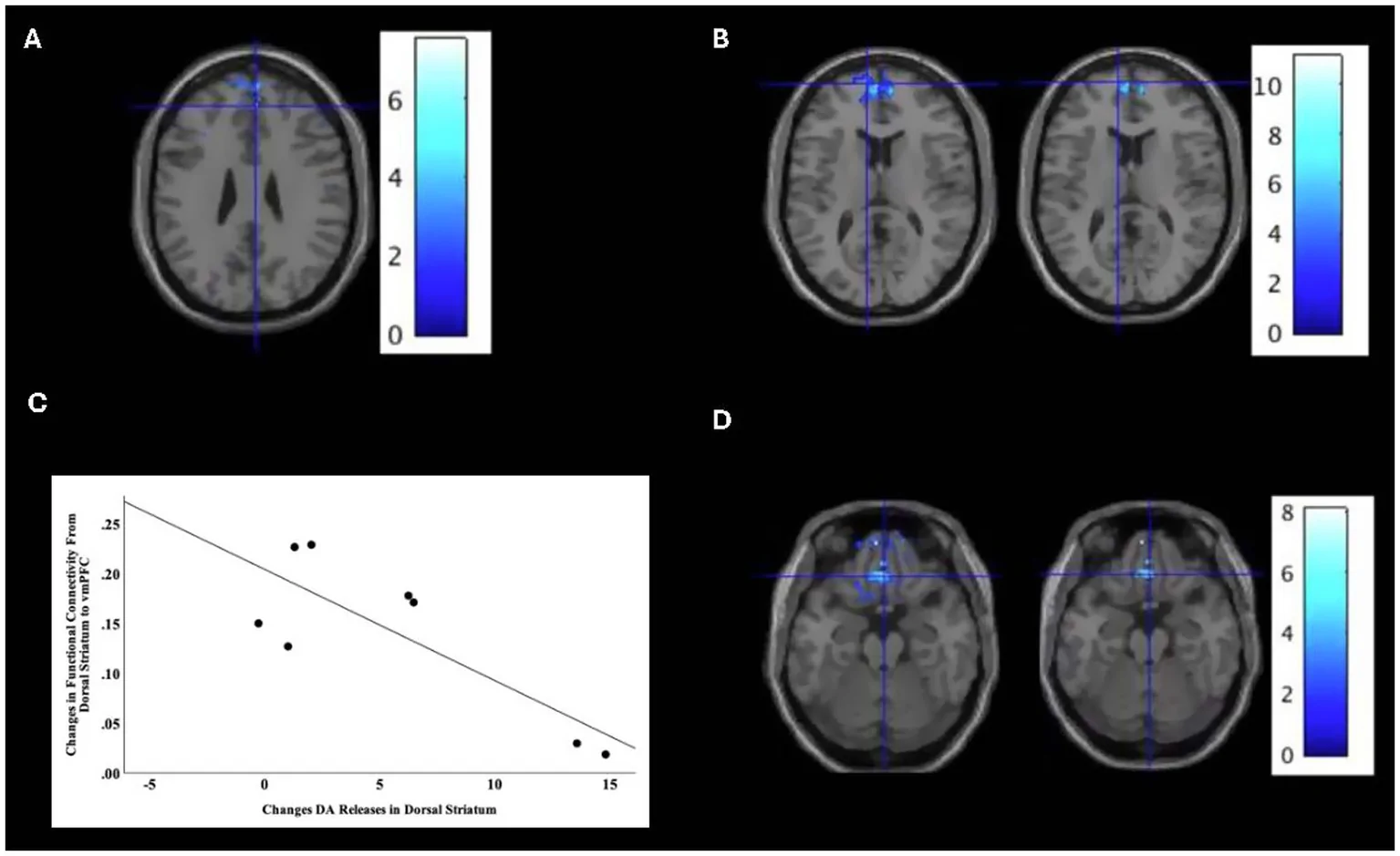
Increased striatal dopamine release is associated with reduced striatal–prefrontal functional connectivity **(A)** Association between striatal dopamine release and striatal–dorsolateral prefrontal cortex functional connectivity. Region-of-interest analysis showing that greater striatal dopamine (DA) release was significantly associated with reduced functional connectivity between the striatum and dorsolateral prefrontal cortex (dlPFC; false discovery rate [FDR]-corrected *p* < 0.05). Crosshairs indicate the peak Montreal Neurological Institute (MNI) coordinates (*x* = 4, *y* = 44, *z* = 28). **(B)** Association between dorsal striatal dopamine release and dorsal striatum–ventromedial prefrontal cortex functional connectivity. Region-of-interest analysis showing that greater dorsal striatal dopamine (DA) release was significantly associated with reduced functional connectivity between the dorsal striatum and ventromedial prefrontal cortex (vmPFC; false discovery rate [FDR]-corrected, *p* < 0.05, the right image). Crosshairs indicate the peak Montreal Neurological Institute (MNI) coordinates (*x* = 2, *y* = 32, *z* = −20). For visualization purposes, the left image is displayed at an uncorrected threshold of *p* < 0.05, whereas the right image displays the FDR-corrected result. **(C)** Association between dorsal striatal dopamine release and dorsal striatum–ventromedial prefrontal cortex functional connectivity. Changes in dorsal striatal dopamine (DA) release were significantly negatively correlated with changes in functional connectivity between the dorsal striatum and ventromedial prefrontal cortex (vmPFC; *r* = −0.809, *p* = 0.015). The solid line represents the fitted linear regression line. **(D)** Association between ventral striatal dopamine release and ventral striatum–ventromedial prefrontal cortex functional connectivity. Region-of-interest analysis showing that greater ventral striatal dopamine (DA) release was significantly associated with reduced functional connectivity between the ventral striatum and ventromedial prefrontal cortex (vmPFC; false discovery rate [FDR]-corrected *p* < 0.05). Crosshairs indicate the peak Montreal Neurological Institute (MNI) coordinates (*x* = −8, *y* = 60, *z* = 12). For visualization purposes, the left image is displayed at an uncorrected threshold of *p* < 0.05, whereas the right image displays the FDR-corrected result.

## 4 Discussion

This study of a limited cohort presents preliminary evidence that the dorsal striatum may serve as a critical hub mediating stress-induced neurochemical and connectivity changes, which could then contribute to addictive behaviors such as smoking. LPS administration induced biological stress and inflammation-related responses, evidenced by elevated cortisol levels compared to PCBO, and increased DA release across all striatal regions. Notably, cortisol elevations *correlated* with dopamine release in the dorsal striatum. Stress also enhanced striatal connectivity to the dlPFC and vmPFC, while elevated dopamine release was negatively associated with these connectivity changes. These preliminary findings point to a potential interaction between stress-induced DA release and prefrontal-striatal connectivity.

Stress-induced cortisol elevations observed in the current study align with findings from our group and others (Zakiniaeiz et al., 2022; Petrulli et al., 2017; Bach et al., 2016). Elevated DA release in the dorsal striatum was correlated with increases in cortisol level. Stress has a profound impact on the dorsal striatum, influencing its roles in motor control, reward-based learning, and habit formation (Balleine et al., 2007; Lipton et al., 2019). Under stress, the neurochemical and functional dynamics of the dorsal striatum could be disrupted, potentially leading to both adaptive and maladaptive behavioral changes. While it is well-established that stress triggers DA release in the striatum (Pruessner et al., 2004), there has been limited direct evidence showing that stress specifically induces DA release in the *dorsal* striatum. Our findings provide preliminary evidence supporting this more targeted observation.

We observed that striatal functional connectivity to the PFC was altered under stress. Specifically, we found that stress increased striatal functional connectivity to the dlPFC and enhanced dorsal striatal functional connectivity to the vmPFC. Our findings are partly in line with several recent studies investigating stress-induced neural mechanisms in stress-related disorders (Seo et al., 2024; Zhukovsky et al., 2024; Leenaerts et al., 2024). They suggest that stress can affect the interactions between the striatal regions and the prefrontal regions, which are critical for executive function and reward processing (Porcelli and Delgado, 2017). The heightened connectivity between the striatum and the dlPFC may reflect an adaptive response aimed at enhancing cognitive control such as decision-making during stress. Conversely, the strengthened connectivity between the dorsal striatum and the vmPFC may indicate a shift toward integrating stress-induced reward-based decision-making processes. Collectively, our preliminary findings highlight the potential dual role of stress in fostering adaptive responses while also increasing the risk of reinforcing maladaptive patterns, particularly those predisposed to addictive behaviors.

On the other hand, we found that stress-induced elevation in DA release in the striatal regions was *negatively* associated with stress-induced changes in striatal functional connectivity. Specifically, elevated DA release in the whole striatum was associated with reduced functional connectivity to the dlPFC, while elevated DA release in the dorsal and ventral striatum were both linked to less connectivity to the vmPFC. To better understand these negative correlations, individual data points were plotted for functional connectivity strength between striatal regions and the PFC, striatal dopamine release, and cortisol levels for both LPS and placebo (PCBO) conditions. The fitting functions used were:

1. y = 1 − exp. (−a * x) + b, where x represents cortisol levels and y represents functional connectivity strength.
2. y = exp. (a * x) + b, where x represents cortisol levels and y represents DA release.

Using the dorsal striatum findings as an example (see Figure 6) for visualization, one can see that DA release in response to stress increases progressively (Figure 6A), whereas stress-induced functional connectivity gradually plateaus once stress reaches a certain level (Figure 6B). The key is to observe the instantaneous slopes of the curves in 6A and 6B within a given stress range. As the rate of change of dopamine release continues to grow, the rate of change of connectivity slows, thus producing a negative correlation between change in DA release and change in connectivity (Figure 6C).

**Figure 6:**
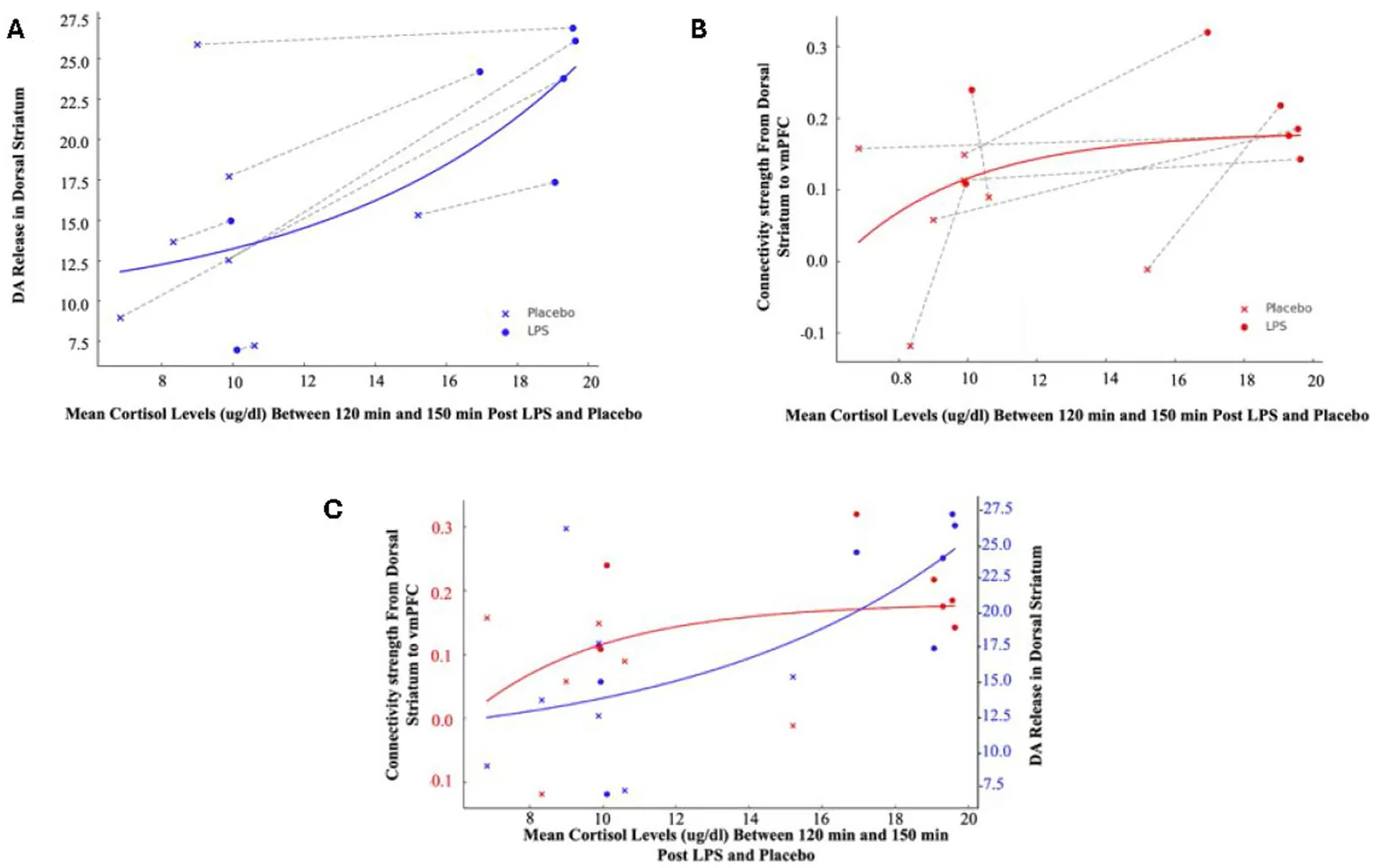
Relationships among changes in cortisol, dorsal striatal dopamine release, and dorsal striatum–vmPFC functional connectivity. **(A)** Relationship between plasma cortisol levels and dorsal striatal dopamine release under LPS and placebo conditions. Mean plasma cortisol levels measured between 120 and 150 min following lipopolysaccharide (LPS) or placebo administration are plotted against dopamine (DA) release in the dorsal striatum. Circles represent the LPS condition, crosses represent the placebo condition, and dashed lines connect observations from the same participant across conditions. The solid line represents the fitted relationship between cortisol levels and dorsal striatal DA release. **(B)** Relationship between plasma cortisol levels and dorsal striatum–ventromedial prefrontal cortex functional connectivity under LPS and placebo conditions. Mean plasma cortisol levels measured between 120- and 150-min following lipopolysaccharide (LPS) or placebo administration are plotted against functional connectivity strength between the dorsal striatum and ventromedial prefrontal cortex (vmPFC). Circles represent the LPS condition, crosses represent the placebo condition, and dashed lines connect observations from the same participant across conditions. The solid line represents the fitted relationship between cortisol levels and dorsal striatum–vmPFC functional connectivity. **(C)** Relationships of plasma cortisol levels with dorsal striatal dopamine release and dorsal striatum–ventromedial prefrontal cortex functional connectivity. Mean plasma cortisol levels measured between 120 and 150 min following lipopolysaccharide (LPS) or placebo administration are plotted against dorsal striatal dopamine (DA) release and functional connectivity between the dorsal striatum and ventromedial prefrontal cortex (vmPFC). Blue symbols and the blue fitted curve represent dorsal striatal DA release, whereas red symbols and the red fitted curve represent dorsal striatum–vmPFC functional connectivity. Circles represent the LPS condition and crosses represent the placebo condition. Both measures are plotted against the same cortisol *x*-axis, with the left *y*-axis representing functional connectivity strength and the right *y*-axis representing dorsal striatal DA release.

Although preliminary, our findings raise the possibility that stress-induced and inflammation related DA release may promote increased functional connectivity within the striatal-prefrontal circuit, which is essential for cognitive control and reward processing (Saint-Cyr, 2003). This could serve as an adaptive mechanism to help the brain cope with stress. However, once stress reaches a certain threshold, this compensatory mechanism may become ineffective, possibly due to a ceiling effect or adaptation to prolonged stress. Importantly, our findings highlight the potential role of the dorsal striatum in stress responses, as cortisol-driven increases in DA release in the dorsal striatum (but not the ventral striatum) were linked to disruptions in dorsal striatal functional connectivity to the vmPFC. Since the vmPFC is not commonly known to project directly to the dorsal striatum, this may indirectly support our findings and further underscore the dorsal striatum’s role as a critical hub in stress-induced neural dysfunction.

A key limitation of the present study is the modest sample size (n = 8), which limits statistical power and generalizability of the findings. Although the within-subject, repeated-measures PET–MRI design substantially reduces inter-individual variability and increases sensitivity to stress-related changes, correlation estimates in small samples are associated with wide confidence intervals and may be sensitive to individual data points. Accordingly, all correlational and voxel-wise findings should be interpreted as preliminary.

With respect to the fMRI component, resting-state functional connectivity findings reflect relative differences between LPS and placebo conditions, rather than absolute pre–post changes from a pure baseline state. As such, the observed connectivity differences should be interpreted accordingly, and future studies incorporating additional baseline or control conditions will be important for delineating stress-specific versus nonspecific effects. More broadly, we acknowledge that the observed neuroimaging effects may reflect inflammatory signaling, stress-related neuroendocrine activation, or their interaction, and these mechanisms cannot be disentangled within the current design.

We acknowledge the risks of multiple regional testing in the context of this small, exploratory sample. While the striatal regions examined were defined *a priori* based on strong theoretical and empirical motivation, the statistically significant findings within hypothesized regions should be interpreted cautiously and viewed as hypothesis-generating.

Finally, while LPS provides a controlled, reproducible, and mechanistically informative model for probing immune–brain interactions relevant to stress, it does not capture all dimensions of psychosocial stress. Future studies integrating both biological (e.g., inflammatory) and psychosocial stress paradigms are desirable for improving generalizability. Because, the sample was predominantly female (6 of 8 participants), there may be sex-related biases in the reported findings. Sex differences in stress responsivity and hypothalamic–pituitary–adrenal (HPA) axis function, dopaminergic signaling, nicotine dependence and smoking behavior, and inflammatory responses to LPS have been well documented. Given the very limited number of male participants, formal analyses of sex differences were obviously not possible. Future studies with larger, more balanced cohorts will be essential to replicate the present results, and directly examine potential sex-dependent mechanisms.

In conclusion, this study offers preliminary insight into the neural pathways through which acute stress could influence DA release in the striatum and alter its communication with prefrontal regions, thus contributing to vulnerability to smoking behaviors. Our findings suggest a distinct role for the dorsal striatum, given that stress-induced cortisol elevations were uniquely associated with increased *dorsal* striatal DA release. Increased DA release was linked to a rise in functional connectivity between the dorsal striatum and the vmPFC, a critical pathway for integrating reward signals and regulating behavior.

## Data Availability

The original contributions presented in the study are included in the article/Supplementary material, further inquiries can be directed to the corresponding author.

## Footnotes

1
https://bioimagesuiteweb.github.io/webapp/

2
https://www.fil.ion.ucl.ac.uk/spm/software/spm12/

3
https://marsbar.sourceforge.net/
