# Supplementary material for: Stress-induced dorsal striatal dopamine release is associated with changes in functional connectivity to the prefrontal cortex in nicotine-dependent individuals—a preliminary report
Source: Front Neurosci. 2026 Oct 27;20:1946892. doi: 10.3389/fnins.2026.1946892 (PMC13760685; doi:10.3389/fnins.2026.1946892)
Supplement: Supplementary file 1 [file Table_1.docx]

| Variable | LPS | narrative | arithmetic | cold press |
| --- | --- | --- | --- | --- |
| temperature | 5, 6, 7, 8, 15, 16, 17, 18 |  |  |  |
| blood pressure | 8 | 11 | 23, 27 | 28 |
| HR | 6, 7, 8, 15, 17 | 19 | 22, 23, 25, 27 | 28, 29** |
| cortisol/corticosterone | 2, 4, 5, 6, 7, 8, 15, 16, 18 | 11 | 20, 21, 22, 24, 26*, 27 | |
| ACTH | 16 | 19 | 22 |  |
| IL-6 | 1, 2, 5, 6, 7, 8, 9, 15, 16, 17, 18 | 31*** | 26*, 30 | 29** |
| IL-10 | 1, 2, 7, 16 |  |  |  |
| TNFa | 1, 2, 5, 6, 7, 8, 9, 16, 17, 18 | 31*** |  |  |
| norepinephrine | 16 |  |  |  |
| Depression/negative emotion up | 2, 8, 9, 15, 17, 18 | 11, 19 | 20, 23, 24, 25, 26*, 27 | |
| positive emotion down | 1, 5, 8 | 11, 19 | 26*, 27 |  |
|  |  |  |  |  |
|  |  |  | **drivers license test...LPS-stim...* | |
|  |  |  | ***LPS-stimulated release* | |
|  |  |  | ****mock job interview* | |

Table S1
